# Maximum Glomerular Diameter and Oxford MEST-C Score in IgA Nephropathy: The Significance of Time-Series Changes in Pseudo-R^2^ Values in Relation to Renal Outcomes

**DOI:** 10.3390/jcm8122105

**Published:** 2019-12-02

**Authors:** Hiroshi Kataoka, Takahito Moriyama, Shun Manabe, Keiko Kawachi, Yusuke Ushio, Saki Watanabe, Taro Akihisa, Shiho Makabe, Masayo Sato, Naomi Iwasa, Yukako Sawara, Mamiko Ohara, Sekiko Taneda, Keiko Uchida, Kazuho Honda, Toshio Mochizuki, Ken Tsuchiya, Kosaku Nitta

**Affiliations:** 1 Department of Nephrology, Tokyo Women’s Medical University, Tokyo 162-8666, Japan; kataoka@twmu.ac.jp (H.K.);; 2 Clinical Research Division for Polycystic Kidney Disease, Department of Nephrology, Tokyo Women’s Medical University, Tokyo 162-8666, Japan; 3 Department of Nephrology, Kameda Medical Center, Chiba 296-8602, Japan; 4 Department of Pathology II, Tokyo Women’s Medical University, Tokyo 162-8666, Japan; 5 Department of Anatomy, Showa University, Tokyo 142-8555, Japan; 6 Department of Blood Purification, Tokyo Women’s Medical University, Tokyo 162-8666, Japan

**Keywords:** immunoglobulin a nephropathy, Oxford MEST-C score, glomerular hypertrophy, prognosis, pseudo-R^2^, renal biopsy

## Abstract

The progression of immunoglobulin A nephropathy (IgAN) is currently assessed using the Oxford MEST-C score, which uses five indicators (mesangial and endocapillary hypercellularity, segmental sclerosis, interstitial fibrosis/tubular atrophy, and the presence of crescents) but has not yet included any risk factors related to glomerular size. Therefore, we tested whether adding another indicator, maximal glomerular diameter (Max GD), would improve the prognostic ability of this scoring system. The data of 101 adult patients diagnosed with IgAN between March 2002 and September 2004 were reviewed. We used McFadden’s pseudo-R^2^ and the corrected Akaike information criterion to assess model fit and the concordance (C)-statistic to assess discriminatory ability. A 10 μm increase in Max GD was significantly associated with a composite outcome (≥50% decline in the estimated glomerular filtration rate or end-stage renal disease). The receiver operating characteristic analysis determined the cut-off for high vs. low Max GD at 245.9 μm, and adding high Max GD to the MEST-C score significantly improved the model’s discrimination of renal outcomes at 5 and ≥10 years. Thus, including the Max GD in the Oxford classification of IgAN might increase its robustness and provide a more comprehensive prognostic system for clinical settings.

## 1. Introduction

Immunoglobulin (Ig) A nephropathy (IgAN) is the most common form of primary glomerulonephritis and a major cause of the end-stage renal disease (ESRD) worldwide [1]. The Oxford classification of IgAN [2,3] was originally introduced to improve individual risk predictions of IgAN progression. However, two major issues required resolution: the low renal prognostic ability of the Oxford classification [4,5] and inconsistency in the renal prognostic power of each marker in the Oxford classification [6,7,8,9]. Other factors may be strongly associated with IgAN progression, and additional clinical markers are required to improve the prediction of renal progression in IgAN [10]. In 2017 [11], in the revised Oxford classification of IgAN, the presence of crescents (C) was added to the conventional histological markers, namely mesangial (M) and endocapillary (E) hypercellularity, segmental sclerosis (S), and interstitial fibrosis/tubular atrophy (T), creating the (MEST-C) scoring system. However, it is unclear whether this scoring system quantitatively improves the prediction of prognosis.

Numerous studies have included various histological and clinical factors to improve renal outcome predictions [5]. For example, a recent study evaluated renal outcome predictors, MEST, renal function, proteinuria, and blood pressure, and reported an R^2^ < 19.1% for a composite outcome comprising an estimated glomerular filtration rate (eGFR) decline of 50% or ESRD [4]. In 2011, we described the maximal glomerular diameter (Max GD), which is an index of the renal corpuscle size, as a new marker for predicting IgAN progression [12]. Although the Max GD was shown to be significantly associated with a ≥1.5-fold increase in the serum creatinine (Cr) level in patients with IgAN, the Oxford classification has not yet included any risk factors related to glomerular size. The present study aimed to confirm the prognostic power of Max GD for renal outcomes and to evaluate whether the prognostic ability of the MEST-C scoring system for patients with IgAN could be improved by adding the Max GD.

## 2. Experimental Section

### 2.1. Study Design 

The present study was approved by the ethics committee at Tokyo Women’s Medical University (No. 5117) and was conducted in accordance with the 1964 Helsinki Declaration and its later amendments or with comparable ethical standards. We reviewed the data of 101 adult patients diagnosed with IgAN between March 2002 and September 2004 at Tokyo Women’s Medical University. Written informed consent for renal biopsy was obtained from all patients for the use of their clinical data at the time of the kidney biopsy; subsequent histological data were obtained from all patients. The inclusion criteria were as follows: a renal biopsy specimen that contained ≥5 glomeruli, on the basis of which three patients were excluded, and the absence of any severe comorbidity, on the basis of which one patient with liver cirrhosis was excluded. Consequently, 97 patients were enrolled in this study (Supplemental Figure S1) and followed until November 2017. To validate the Oxford classification study, 84 patients were examined whose renal biopsy specimens contained ≥8 glomeruli. Supplemental Material S1 describes the measurement of covariates, definitions of comorbidities, and the histological assessment of kidney biopsies.

### 2.2. Pathological Analyses

All kidney tissue specimens were obtained through percutaneous needle biopsies. The specimens were fixed in 10% phosphate-buffered formalin (pH 7.2), embedded in paraffin wax, and cut into 4-µm sections. The sections were stained with hematoxylin and eosin, periodic acid-Schiff, silver methenamine, or Masson’s trichrome for light microscopy. Each specimen was evaluated for glomerular, interstitial, and vascular changes [12]. The Max GD was calculated as the mean of the maximal diameter of the glomerulus and the maximal chord perpendicular to the maximal diameter of the maximally hypertrophied glomerulus (the largest renal corpuscle) in the area with the maximal profile in each specimen [12,13].

A validation study of the Oxford classification included 84 patients who met Oxford criteria [2,3,11], which required biopsies containing ≥8 glomeruli. The MEST-C criteria comprised mesangial hypercellularity (with M0 and M1 corresponding to ≤50% and >50% of the glomeruli showing hypercellularity, respectively), endocapillary hypercellularity (defined as E0: absent or E1: present), segmental glomerulosclerosis (defined as S0: absent or S1: present), tubular atrophy/interstitial fibrosis (with T0, T1, and T2 corresponding to ≤25%, 26%–50%, and >50% of cortical area involvement, respectively), cellular/fibro cellular crescents (with C0, C1, and C2 corresponding to their absence, presence in ≥1 and <25% of glomeruli, and presence in ≥25% of the glomeruli, respectively). 

### 2.3. Statistical Analyses

Continuous variables were reported as means and standard deviations or as medians (minimum-maximum). Categorical variables were reported as percentages unless otherwise stated. Group differences were evaluated using the unpaired t-test, Mann–Whitney U test, Chi-square test, or Fisher’s exact test, as appropriate. The prognostic variables for renal outcomes were assessed using univariate and multivariate Cox proportional hazards models. Variables with *p*-values <0.1 in the univariate model, as well as age, sex, and eGFR, were included in the multivariate model. The optimal Max GD cut-off value for discriminating an eGFR decline ≥50% or ESRD during follow-up was determined by a receiver operating characteristic (ROC) analysis. Based on the ROC analysis, we divided the patients into two groups, namely the high Max GD group (Max GD ≥245.9 μm) and the low Max GD group (Max GD <245.9 μm). Survival curves were plotted using the Kaplan–Meier method and evaluated using the log-rank test. To reduce confounding biases, we fitted propensity score-matched models that included potentially modifying variables, namely, age, mean blood pressure (MBP), and eGFR; additionally, we performed subgroup analyses. The caliper-matching method was used, with a maximum tolerance level of 0.1.

To validate the Oxford classification, components of the MEST-C score with and without large renal corpuscles (Max GD ≥245.9 μm) were considered. Model discriminatory ability was evaluated using the concordance (C)-statistic [14,15], and model goodness of fit was assessed using McFadden’s pseudo-R-squared (pseudo-R^2^) [16] and the corrected Akaike information criterion (AICc) [17]. All statistical tests were 2-tailed, and *p* < 0.05 was considered statistically significant. Statistical analyses were performed using JMP Pro software, version 14.1.0 (SAS Institute, Cary, NC, USA). 

## 3. Results

### 3.1. Patients’ Characteristics

Ninety-seven patients (42 men and 55 women; mean age at the time of renal biopsy, 34 ± 12.6 years) met the study’s inclusion criteria (Supplemental Figure S1). The MBP was 91.6 ± 13 mmHg, median proteinuria level was 0.72 g/day (0–4.20 g/day), and mean eGFR was 71.2 ± 19.7 mL/min/1.73 m^2^ (Table 1). Of the 97 patients, 55 received corticosteroids, and 69 received angiotensin-converting enzyme inhibitors or angiotensin receptor blockers during follow-up. The median follow-up duration was 11.9 years.

### 3.2. Patients’ Pathological Features

The median number of glomeruli examined per subject was 13 (5–46). The global glomerulosclerosis rate was 11.1 (0%–75%). The mean Max GD was 218.3 ± 27 μm. The percentages of patients in our Oxford classification validation cohort with a Max GD ≥245.9 μm were 47.6%, 55.9%, 86.9%, 16.7%, 2.4%, 46.4%, 10.7%, and 20.2% for M1, E1, S1, T1, T2, C1, C2, and Max GD ≥245.9 μm respectively (Table 2). 

### 3.3. A High Max GD as a Prognostic Indicator 

**Figure 1:**
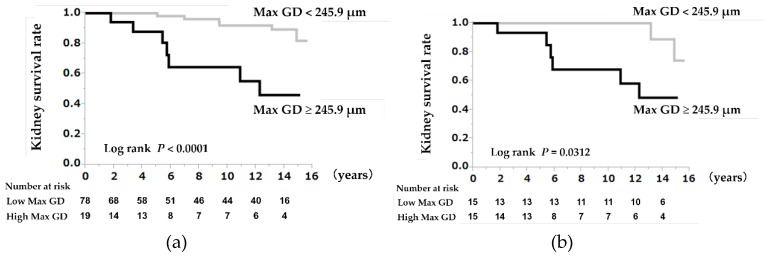
(**a**) Kidney survival rates in the high Max GD group (Max GD ≥ 245.9 μm) and low Max GD group (Max GD < 245.9 μm) within the entire cohort. The renal prognosis for patients with large renal corpuscles (glomerular hypertrophy) with Max GD ≥ 245.9 μm was poor. (**b**) Kidney survival rate in the high Max GD group (Max GD ≥ 245.9 μm) and the low Max GD group (Max GD < 245.9 μm) in the propensity score-matched cohort. The renal prognosis for patients with large renal corpuscles (glomerular hypertrophy) and Max GD ≥ 245.9 μm was poor after matching the groups in terms of age, MBP, and eGFR. Abbreviations: Max GD, maximal glomerular diameter; MBP, mean blood pressure; eGFR, estimated glomerular filtration rate.

Multivariate Cox regression analyses showed that an eGFR decline ≥50% or ESRD was significantly associated with a 10-µm increase in the Max GD (hazard ratio (HR) = 1.51, 95% confidence interval (CI) 1.08–1.67, *p* = 0.0192) (Table 3). Kaplan–Meier analyses showed that the kidney survival rate in the high Max GD group (≥245.9 μm) was significantly lower than that in the low Max GD group (<245.9 μm) (log-rank *p* < 0.0001) (Figure 1a).

### 3.4. Clinical and Pathological Findings According to the Max GD Value

Comparative analyses revealed that the patients with a high Max GD were older, and their blood pressure, serum Cr, triglyceride, and urinary β2-microglobulin levels, IgA, complement component (C) 4, and IgA/C3 ratios were higher, and their eGFRs were lower than those in the patients with a low Max GD. The hyperuricemia ratio as comorbidity and the ratio between immunosuppressive agent use and calcium-channel blocker use were higher in the high Max GD group than in the low Max GD group (Table 1). Histologically, interstitial fibrosis, arteriosclerosis, and arteriolar hyalinosis levels were higher in the high Max GD group than in the low Max GD group (Table 2).

### 3.5. A High Max GD as a Prognostic Indicator in the Propensity Score-Matched Cohorts

The results of the propensity score-matched models and subgroup analyses of the high Max GD (257.3 ± 7.8 µm) and low Max GD (206.1 ± 18 µm) groups (*p* < 0.0001) are provided in Supplemental Tables S1 and S2. The Kaplan–Meier analysis, with an eGFR decline ≥50% or ESRD as the endpoint, revealed that the kidney survival rate was significantly lower in the high Max GD group than in the low Max GD group, after adjusting for the eGFR (log-rank *p* = 0.0312) (Figure 1b).

### 3.6. Validation of the Prognostic Values of the MEST-C Score and Max GD

**Figure 2:**
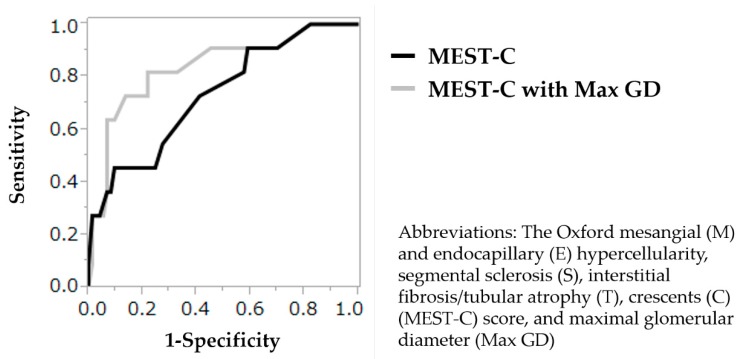
Receiver operating characteristic curves and the C-statistic (area under the curve) for models predicting the risk of an estimated glomerular filtration rate decline ≥50% or end-stage renal disease using the Oxford MEST-C score with and without Max GD ≥245.9 μm. Adding Max GD ≥245.9 μm to the MEST-C score significantly improved discrimination regarding renal outcomes, as measured by the change in the C-statistic from 0.733 to 0.837. Abbreviations: Max GD, maximal glomerular diameter.

Adding Max GD ≥245.9 μm to the MEST-C score improved the renal outcome prediction compared to that observed using the MEST-C score alone. Adding the Max GD to the MEST-C score increased the C-statistic from 0.733 (for the MEST-C score alone) to 0.837, which significantly improved the model’s discriminatory ability to predict the renal outcome after biopsy (Figure 2), increased the McFadden’s pseudo-R^2^ value by 0.093 (from 0.159 to 0.252), and reduced the AICc by 3.7 (from 67.9 to 64.2).

### 3.7. Time-Series Change in Pseudo-R^2^ Values of the Prognostic Efficacy in Relation to Renal Outcomes

**Figure 3:**
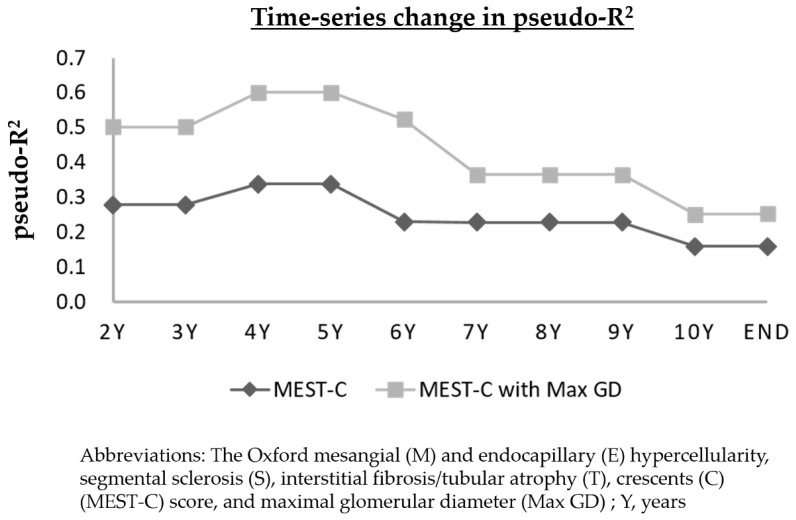
Time-series change in pseudo-R^2^ values of the prognostic efficacy in relation to renal outcomes. The lower line represents the time-series change in the pseudo-R^2^ values for the Oxford MEST-C score, and the upper line shows the time-series change in the pseudo-R^2^ value of the Oxford MEST-C score with a Max GD ≥ 245.9 μm. Adding Max GD ≥ 245.9 μm to the Oxford-MEST-C score improved the model’s ability to predict the risk of eGFR decline ≥ 50% or end-stage renal disease by about 2-fold in the short-term (2–6 years) and by about 1.5-fold in the long-term (from 7 years until the end of the study). Abbreviations: eGFR, estimated glomerular filtration rate

**Figure 4:**
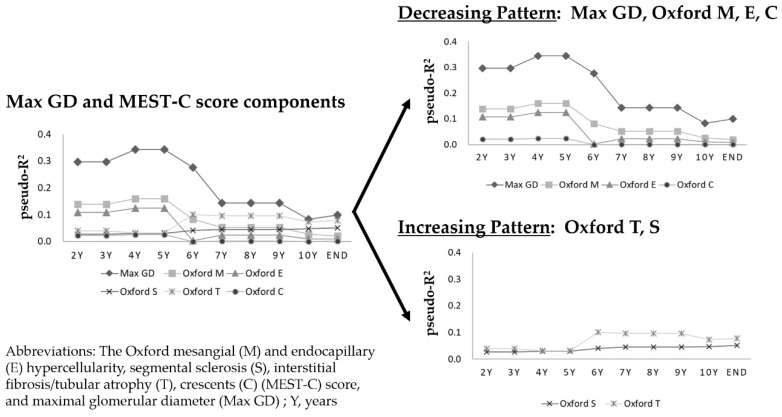
Time-series change in pseudo-R^2^ values of prognostic efficacy in relation to renal outcomes: Max GD and individual Oxford MEST-C score components. Two patterns of change in pseudo-R^2^ values emerged for the Max GD and individual MEST-C score components in the time-series analyses. For short-term prognostic predictions, the Max GD, Oxford M, and Oxford E components were key factors. Although the prognostic potential of the Max GD tended to decrease after 6 years, it had the greatest predictive power with respect to the renal prognosis after ≥10 years.

Time-series change in the pseudo-R^2^ values of the Max GD, individual components of the MEST-C score, and the sum of the MEST-C score with and without the Max GD are shown in Table 4 and Figure 3 and Figure 4. The pseudo-R^2^ value for the MEST-C score with the Max GD peaked at 0.6011 at 4 and 5 years after kidney biopsy and fell to 0.2523 at the end of the follow-up period (Figure 3). The pseudo-R^2^ values for the MEST-C score with a Max GD ≥245.9 μm were higher than those for the MEST-C score alone at all follow-up intervals.

Two patterns of change in the pseudo-R^2^ values emerged for the Max GD and individual MEST-C score components in the time-series analyses. The pseudo-R^2^ values declined when the Max GD and Oxford M, E, and C components were included, and they increased when the Oxford S and T components were included. For example, the pseudo-R^2^ value for the Max GD was >0.3 (maximum, 0.3438) at 4 and 5 years after a kidney biopsy, and although the Max GD pseudo-R^2^ value remained the highest among all examined variables, it declined gradually to <0.1 by the end of the study. In contrast, the pseudo-R^2^ value for the Oxford T was <0.04 at 2–5 years, and it gradually increased to >0.07 by the end of the study (Figure 4).

## 4. Discussion

The present study’s two main findings were as follows: the renal prognostic ability of the Oxford score for IgAN was significantly improved by adding the Max GD, which is a new index of renal pathology, and two patterns of change in the pseudo-R^2^ values were revealed in the time-series analyses of renal prognosis prediction. These new findings could strongly influence clinical practice.

Glomerular hypertrophy plays a crucial role in kidney disease outcomes in experimental models [18,19,20,21] and humans [22,23,24]. Large renal corpuscles are easily measured and quantified in kidney biopsy specimens. However, the prognostic potential of large renal corpuscles has yet to be fully realized in clinical settings. While several explanations are available for this lack of the adoption of renal corpuscle measurement in clinical settings [25], the most meaningful explanation is the lack of consensus regarding how to account for sclerosing and collapsing glomeruli. As reported previously [26], some injured glomeruli increase in size before they sclerose and collapse, and glomerular hypertrophy precedes glomerulosclerosis. Consequently, renal corpuscles of different sizes, including hypertrophied and collapsing glomeruli, are present within the same kidney specimen. As sclerotic and collapsing glomeruli have the same size as that of normal glomeruli [12], measuring the maximal glomerular size facilitates the examination of the significance of the renal corpuscle size [25]. 

The Max GD, rather than the glomerular tuft size, can be used to indicate the renal corpuscle size, as it includes the area occupied by Bowman’s space, which itself could be significant pathophysiologically [27]. Additionally, the morphology of the renal corpuscle (Bowman’s capsule) is less susceptible to sclerosis and collapse than that of the glomerular tuft. Furthermore, the renal corpuscle is easier to measure than is the glomerular tuft. For a reproducible measurement, the most important procedure is to draw the maximal diameter that passes through the geometric center of the maximal profile of the glomerulus. The position of the geometric center of the maximal profile of the glomerulus is identifiable visually. After drawing the maximal diameter that passes through the geometric center, we draw the maximal chord perpendicular to the maximal diameter. The Max GD is calculated as the mean of these. In the present study, the Max GD cut-off value was determined to be 245.9 μm, which was similar to that reported in our previous study (242.3 μm). We considered a pathological threshold to exist for glomerular size, discriminating between morbid glomerular and physiological glomerular hypertrophy [25]. A large renal corpuscle is a marker of a low eGFR, the original disease activity marker, and reflects impairment in different metabolic risk states [25]. In the present study, a high Max GD was associated with numerous factors, namely, blood pressure; serum triglyceride, IgA, C4, and urinary β2-microglobulin levels; IgA/C3 ratio; ratio of hyperuricemia as a comorbidity; and ratio between immunosuppressant use and calcium-channel blocker use, which were higher in the high Max GD group than in the low Max GD group. Although age and glomerular loss cannot be modified, it is possible to correct hypertension, hypertriglyceridemia, hyperuricemia, high serum IgA and C4 levels, and a high serum IgA/C3 ratio. Hence, therapy may be planned based on the presence of large renal corpuscles, thereby improving renal outcomes.

While the multifactorial characteristics of the Max GD may provide useful information regarding patient treatment, the focus may be shifted away from the diagnostic classifications of IgAN, including the Oxford classification. However, the Oxford classification was introduced originally to improve the individualized risk prediction of IgAN progression [2,3]. Although the renal prognostic ability of the Oxford classification of IgAN was lower than expected in clinical settings, the present study demonstrated that adding the Max GD to the MEST-C score significantly improved its prognostic ability. 

We evaluated the change in the pseudo-R^2^ values over time because both short-term and long-term renal prognoses are clinically relevant [4,28]. The present study was the first to show the time-series change in pseudo-R^2^ values in relation to the prognostic abilities of renal pathological factors. The findings of the present study suggested that the MEST-C score combined with a Max GD ≥245.9 μm might explain the 60.1% decline in the eGFR or ESRD at 4 and 5 years after kidney biopsy and that the Max GD improved the model’s short-term renal prognostic ability by 34.4% for patients with IgAN. By adding the Max GD to the Oxford MEST-C score, the prediction of short-term renal prognoses improved 2-fold and that for the long-term renal prognoses improved 1.5-fold. 

More interestingly, we found two patterns of time-series change in the pseudo-R^2^ values of the Max GD and individual components of the MEST-C score. The pseudo-R^2^ values declined over time when the Max GD and Oxford M, E, and C components were evaluated, and they increased over time when the Oxford S and T components were evaluated. Time-series change in pseudo-R^2^ values has not been previously evaluated in the study of kidney diseases; thus, the present study results raised questions regarding the causes underlying the different patterns of change over time. Regarding the Oxford MEST-C score, Barbour et al. [4] examined early risk predictions in patients with IgAN and showed that the presence of M1 was a histological marker that predicted the benefits of steroid therapy. Chakera et al. [29] reported that E1 was an independent predictor of the rate of loss of renal function in patients with IgAN who did not receive any immunosuppression. Haas et al. [30] found that crescents predicted an eGFR decline ≥50% or ESRD in patients with IgAN who did not receive any immunosuppression, and this finding supported the addition of the C0, C1, and C2 scores to the Oxford MEST score. In contrast, Coppo et al. [28] recently examined the long-term implications of the MEST score in IgAN, and found that M1, S1, and T1–T2 lesions were independently related to the renal outcome in their entire cohort; the HRs determined in multivariable Cox regression analyses were highest (2.46) for Oxford T1–T2, and second-highest (1.61) for Oxford S1. Hence, the Oxford M, E, and C components are therapeutically reactive and may be susceptible to changes in prognostic ability. The Oxford S and T components may include irreversible damage and subsequent poor therapeutic responses. Therefore, the Oxford M, E, and C components are likely to affect short-term renal prognoses, and the Oxford S and T components are likely to affect long-term renal prognoses. We considered that the two patterns of time-series change in pseudo R^2^ values might reflect these characteristics of the Oxford MEST-C score. Several investigators who retrospectively studied the Oxford MEST-C classification of IgAN confirmed the high prognostic relevance of the Oxford S and T components [6,7,8,9,31,32,33]; however, differences regarding treatment, outcome measures, and patient selection criteria are thought to cause inconsistencies regarding the predictive values of the M, E, and C components. The present findings might help resolve these inconsistencies. Therefore, when considering renal prognoses using renal histological parameters, awareness of the long-term and short-term prognoses is critical for time-series forecasting.

The relationships between the Max GD pseudo-R^2^ value and short-term and long-term renal prognoses suggested that Max GD represented a variety of pathological conditions, including immunological inflammation, lifestyle-related diseases, and irreversible damage. Short-term renal prognoses are influenced by inflammation and lifestyle-related diseases, and long-term renal prognoses are influenced by irreversible lesions and lifestyle-related diseases, including atherosclerosis. The present study results suggested it might be possible to improve the renal prognosis in patients with IgAN and large renal corpuscles by administering treatment regimens that address the Oxford M, E, or C components, such as immunosuppressive agents, as these components also showed declines in their pseudo-R^2^ values. Furthermore, treatments, including antihypertensive, antidyslipidemic, and antihyperuricemic agents, might be tailored to a patient’s clinical status. In this context, Max GD might respond to a variety of pathophysiological injuries associated with IgAN, which suggests that it is an ideal indicator of IgAN progression.

While these findings might have broader implications for patients with all kidney diseases, our study had several limitations. First, only the patients’ baseline characteristics were considered; their characteristics during the follow-up period were not considered. Second, the study was observational, so the observed associations do not prove causality. Third, the sample size was relatively small; hence, further studies are required to confirm the present findings in a large patient cohort. Fourth, this study only enrolled Japanese patients from a single center. Some clinical characteristics of our population were somewhat different from those of typical white patients with IgAN, such as a lower prevalence of obesity. Therefore, we need to confirm our findings in other ethnic groups. Fifth, during the long-term follow-up period, patients’ treatments could be changed according to the progression of kidney disease. However, these changes were not reflected in this analysis.

## 5. Conclusions

The Max GD, which is easily quantified histologically in needle biopsies, could be used as a prognostic indicator of IgAN progression. Adding a Max GD ≥245.9 μm to the Oxford MEST-C score significantly improved the short-term and long-term predictions of renal outcomes in patients with IgAN. Time-series change in the pseudo-R^2^ values for the Max GD produced data that were highly suggestive of renal progression in IgAN. Including the Max GD in the Oxford classification of IgAN might increase its robustness and provide a more comprehensive prognostic system for clinical settings.

## Figures and Tables

**Table 1:** Clinical and laboratory findings; Entire cohort, n = 97.

Variables	Entire Cohort	Max GD ≥245.9 μm	Max GD <245.9 μm	*p*-Value	Standardized Differences
	n = 97	n = 19	n = 78		
Clinical Findings					
Age (years)	34 ± 12.6	42.3 ± 15.3	32 ± 11.1	0.0013	0.771
Sex (Male; n (%))	42 (43.3)	11 (57.9)	31 (39.7)	0.1985	0.370
BMI (kg/m^2^)	22.2 ± 3.1	21.8 ± 2.4	22.2 ± 3.2	0.6172	0.141
SBP (mmHg)	122.6 ± 16.3	130.6 ± 21	120.7 ± 14.4	0.0163	0.550
DBP (mmHg)	76.1 ± 12.8	81.2 ± 14.3	74.8 ± 12.1	0.0494	0.483
MBP (mmHg)	91.6 ± 13	97.7 ± 15.8	90.1 ± 11.9	0.0224	0.543
PP (mmHg)	46.5 ± 10.9	49.4 ± 12.2	45.8 ± 10.6	0.2099	0.315
Laboratory Findings					
Total protein (g/dL)	6.63 ± 0.63	6.71 ± 0.84	6.61 ± 0.57	0.5423	0.139
Serum albumin (g/dL)	3.93 ± 0.41	3.84 ± 0.48	3.95 ± 0.39	0.3101	0.252
Blood urea nitrogen (mg/dL)	15.5 ± 6.1	17.8 ± 5.7	14.9 ± 6.1	0.0736	0.491
Serum creatinine (mg/dL)	0.90 ± 0.29	1.07 ± 0.31	0.86 ± 0.27	0.0041	0.722
eGFR (mL/min/1.73 m^2^)	71.2 ± 19.7	58 ± 18	74.4 ± 18.9	0.0009	0.889
Uric acid (mg/dL)	5.76 ± 1.73	6.29 ± 1.65	5.63 ± 1.73	0.1478	0.390
Total cholesterol (mg/dL)	192.1 ± 37.5	197.8 ± 31.8	190.7 ± 38.8	0.4653	0.200
Triglyceride (mg/dL)	119.2 ± 70	154.7 ± 94.9	110.6 ± 60.2	0.0130	0.555
Hemoglobin A1c (NGSP) (%)	5.39 ± 0.41	5.53 ± 0.30	5.35 ± 0.42	0.1220	0.493
IgG (mg/dL)	1131.6 ± 239.9	1158.4 ± 281.4	1125.1 ± 230.3	0.5900	0.130
IgA (mg/dL)	314.2 ± 110.9	387.4 ± 145.3	296.4 ± 93.6	0.0011	0.745
IgM (mg/dL)	119.7 ± 53.9	108.8 ± 53.1	122.4 ± 54.1	0.3270	0.254
CH50 (mg/dL)	39.5 ± 8.4	40.6 ± 8.8	39.2 ± 8.3	0.5137	0.164
C3 (mg/dL)	97.1 ± 16.8	102 ± 22.2	95.9 ± 15.2	0.1531	0.321
C4 (mg/dL)	22.8 ± 6.3	26.3 ± 5.7	22 ± 6.2	0.0067	0.722
IgA/C3 ratio	3.31 ± 1.13	3.86 ± 1.49	3.18 ± 0.99	0.0174	0.538
U-Prot (g/day)	0.72 (0–4.20)	0.78 (0–4.20)	0.70 (0–2.74)	0.1496	0.427
U-RBC (counts/HPF)	20 (0–100)	10 (1–100)	20 (0–100)	0.2638	0.153
U-NAG (U/g·Cre)	5.9 (1.7–25)	5.9 (3.4–22.1)	5.9 (1.7–25)	0.2769	0.352
U-β2MG (µg/g·Cre)	100 (0–3464.4)	302.2 (17.7–3464.4)	98.4 (0–1223.2)	0.0197	0.737
Initial treatments					
Corticosteroids (n (%))	55 (57.3)	10 (52.6)	45 (58.4)	0.7964	0.117
Tonsillectomy (n (%))	24 (25)	3 (15.8)	21 (27.3)	0.3852	0.282
Corticosteroids combined with tonsillectomy (n (%))	18 (18.8)	2 (10.5)	16 (20.8)	0.5120	0.286
Immunosuppressants (n (%))	2 (2.1)	2 (10.5)	0 (0)	0.0375	0.484
Concomitant drugs					
Antihypertensive agents (n (%))	70 (72.9)	16 (84.2)	54 (70.1)	0.2623	0.341
ARB and or ACEI (n (%))	69 (71.9)	16 (84.2)	53 (68.8)	0.2574	0.369
CCB (n (%))	18 (18.8)	7 (36.8)	11 (14.3)	0.0439	0.534
Anti-platelet agents	61 (63.5)	13 (68.4)	48 (62.3)	0.7914	0.128
Anti-coagulation	5 (5.2)	2 (10.5)	3 (3.9)	0.2561	0.257
EPA (n (%))	38 (39.6)	8 (42.1)	30 (39)	0.7996	0.063
No therapy (n (%))	6 (6.3)	0 (0)	6 (7.8)	0.5951	0.411
Comorbidities					
Hypertension (n (%))	71 (74)	16 (84.2)	55 (71.4)	0.3829	0.312
Hyperuricemia (n (%))	39 (41.5)	12 (63.2)	27 (36)	0.0394	0.565
Hypertriglyceridemia (n (%))	38 (39.6)	10 (52.6)	28 (36.4)	0.2040	0.330
Hypercholesterolemia (n (%))	29 (30.2)	8 (42.1)	21 (27.3)	0.2654	0.315

Continuous variables were expressed as means ± standard deviation or median (minimum-maximum). Count data were expressed as n (%). Abbreviations: n, number; %, percentages; Max GD, maximal glomerular diameter; BMI, body mass index; SBP, systolic blood pressure; DBP, diastolic blood pressure; MBP, mean blood pressure; PP, pulse pressure; eGFR, estimated glomerular filtration rate; IgG, immunoglobulin G; IgA, immunoglobulin A; IgM, immunoglobulin M; CH50, 50% hemolytic complement activity; C3, complement component 3; C4, complement component 4; U-Prot, Urinary protein excretion; U-RBC, urinary red blood cells; HPF, high-power field; U-NAG, urinary N-acetyl-beta-D-glucosaminidase; U-β2MG, urinary β2-microglobulin; Cre, creatine; ARB, angiotensin receptor blocker; ACEI, angiotensin-converting enzyme inhibitor; CCB, calcium-channel blocker; EPA, eicosapentaenoic acid.

**Table 2:** Histological findings; Entire cohort, n = 97.

Variables	Entire Cohort	Max GD ≥245.9 μm	Max GD <245.9 μm	*p*-Value	Standardized Differences
	n = 97	n = 19	n = 78		
Number of glomeruli	13 (5–46)	13 (7–46)	13 (5–36)	0.1988	0.441
Global sclerosis (%)	11.1 (0–75)	13.9 (0–50)	11.1 (0–75)	0.1160	0.339
Segmental sclerosis or adhesion (%)	12.5 (0–83.3)	20 (0–44.4)	12.5 (0–83.3)	0.2223	0.176
Segmental sclerosis (%)	0 (0–37.5)	0 (0–28.6)	0 (0–37.5)	0.8250	0.020
Adhesion (%)	12.5 (0–83.3)	16.7 (0–44.4)	12.5 (0–83.3)	0.8172	0.036
Crescent (%)	8.3 (0–55.6)	14.3 (0–44.4)	7.1 (0–55.6)	0.3351	0.139
Cellular or Fibro-cellular (%)	3.9 (0–55.6)	8.3 (0–33.3)	0 (0–55.6)	0.2016	0.179
Fibrous (%)	0 (0–24)	0 (0–11.1)	0 (0–24)	0.4035	0.155
Mesangial cell proliferation (0–3)	1 (0–3)	2 (0–2)	1 (0–3)	0.0598	0.396
Mesangial matrix expansion (0–3)	1 (0–3)	1.5 (0–2)	1 (0–3)	0.5536	0.070
Interstitial fibrosis (%)	14.5 ± 11.5	20.1 ± 10.3	13.2 ± 11.4	0.0172	0.635
Interstitial fibrosis (0–3)	1 (0–3)	1 (1–2)	1 (0–3)	0.0030	0.816
Interstitial inflammation (0–3)	1 (0–2)	1 (0–2)	1 (0–2)	0.2011	0.300
Arteriosclerosis (0–3)	0 (0–2)	1 (0–2)	0 (0–2)	0.0324	0.597
Arteriolar hyalinosis (0–3)	0 (0–3)	1 (0–3)	0 (0–3)	0.0268	0.580
Max GD (µm)	218.3 ± 27	258.4 ± 8.5	208.6 ± 20	<0.0001	3.241
Oxford Classification (n = 84)					
M1	40 (47.6)	11 (64.7)	29 (43.3)	0.1735	0.440
E1	47 (55.9)	10 (58.8)	37 (55.2)	1	0.073
S1	73 (86.9)	16 (94.1)	57 (85.1)	0.4481	0.298
T1	14 (16.7)	5 (29.4)	9 (13.4)	0.1458	0.398
T2	2 (2.4)	0 (0)	2 (3)	1	0.249
C1	39 (46.4)	10 (58.8)	29 (43.3)	0.2861	0.314
C2	9 (10.7)	2 (11.8)	7 (10.5)	1	0.041

Continuous variables were expressed as means ± standard deviation or median (minimum-maximum). Count data were expressed as n (%). Abbreviations: n, number; %, percentages; Max GD, maximal glomerular diameter; M, mesangial hypercellularity; E, endocapillary hypercellularity; S, segmental glomerulosclerosis; T, tubular atrophy/interstitial fibrosis; C, cellular/fibro cellular crescents.

**Table 3:** Univariate and multivariate analysis of risk factors associated with a ≥50% eGFR decline or ESRD (Entire cohort, n = 97).

Variables	Univariate Analysis		Multivariate Analysis	
	Hazard Ratio (95% CI)	*p*-Value	Hazard Ratio (95% CI)	*p*-Value
Clinical and Laboratory Findings				
Age (10-year increase)	2.01 (1.29–3.07)	0.0027	0.73 (0.18–2.99)	0.6513
Men (vs. women)	1.84 (0.61–5.73)	0.2741	4.86 (0.62–41.58)	0.1250
BMI (1 kg/m^2^ increase)	1.10 (0.94–1.25)	0.2181	-	-
MBP (10 mmHg increase)	1.82 (1.18–2.86)	0.0068	0.56 (0.17–1.65)	0.2798
eGFR (10 mL/min/1.73 m^2^ increase)	0.45 (0.31–0.64)	<0.0001	0.43 (0.19–0.81)	0.0085
Hemoglobin (1 g/dL increase)	0.95 (0.67–1.32)	0.7849	-	-
Serum albumin (1 g/dL increase)	0.18 (0.06–0.57)	0.0041	0.02 (0–0.49)	0.0175
U-Prot (g/day)	1.86 (1.16–2.79)	0.0117	0.44 (0.12–1.68)	0.2128
Hypercholesterolemia (vs. no)	3.68 (1.22–12.21)	0.0206	1.50 (0.03–74.43)	0.8424
Hypertriglyceridemia (vs. no)	3.78 (1.23–13.99)	0.0199	7.90 (0.16–568.61)	0.3407
Hyperuricemia (vs. no)	6.94 (2.11–31.01)	0.0011	2.71 (0.27–45.47)	0.4123
Initial treatments				
Corticosteroids (vs. no)	0.54 (0.18–1.64)	0.2763	-	-
Tonsillectomy (vs. no)	0.36 (0.06–1.35)	0.1411	-	-
Corticosteroids combined with tonsillectomy (vs. no)	0.60 (0.09–2.24)	0.4851	-	-
Immunosuppressants (vs. no)	10.25 (0.53–64.22)	0.1009	-	-
Histological findings				
Global sclerosis (%)	1.04 (1.01–1.07)	0.0227	0.93 (0.85–0.99)	0.0434
Segmental sclerosis or adhesion (%)	1.01 (0.98–1.04)	0.5240	-	-
Segmental sclerosis (%)	1.03 (0.97–1.07)	0.2967	-	-
Adhesion (%)	0.99 (0.95–1.02)	0.5415	-	-
Crescent (%)	1 (0.95–1.03)	0.9028	-	-
Cellular or Fibro-cellular (%)	0.99 (0.94–1.04)	0.8018	-	-
Fibrous (%)	0.99 (0.86–1.08)	0.8393	-	-
Mesangial cell proliferation (0–3)	2.26 (0.84–6.28)	0.1086	-	-
Mesangial matrix expansion (0–3)	3.07 (1.29–7.29)	0.0119	0.38 (0.04–2.95)	0.3684
Interstitial fibrosis (0–3)	6.06 (2.46–16.17)	<0.0001	10.97 (1.36–129.18)	0.0242
Interstitial inflammation (0–3)	1.94 (0.80–4.70)	0.1431	-	-
Arteriosclerosis (0–3)	2.40 (1.25–4.84)	0.0085	3.10 (0.70–16.38)	0.1354
Arteriolar hyalinosis (0–3)	2.78 (1.34–6.07)	0.0064	0.79 (0.08–7.67)	0.8348
Max GD (10 µm increase)	1.37 (1.08–1.67)	0.0069	1.51 (1.08–2.36)	0.0192
Oxford Classification (n = 84)				
M0/M1	2.24 (0.75–7.42)	0.1498	NA	-
E0/E1	0.66 (0.20–1.99)	0.4662	NA	-
S0/S1	1.33 (0.41–5.96)	0.6566	NA	-
T0/T1/T2	4.54 (1.74–11.20)	0.0031	NA	-
C0/C1/C2	1.21 (0.50–2.72)	0.6636	NA	-

Variables with *p*-values of less than 0.1 in the univariate model, age, sex, and eGFR were included in the multivariate model. Abbreviations: eGFR, estimated glomerular filtration rate; ESRD, end-stage renal disease; n, number; %, percentages; CI = confidence interval; vs, versus; BMI, body mass index; MBP, mean blood pressure; U-Prot, Urinary protein excretion; Max GD, maximal glomerular diameter; NA, not applicable; M, mesangial hypercellularity; E, endocapillary hypercellularity; S, segmental glomerulosclerosis; T, tubular atrophy/interstitial fibrosis; C, cellular/fibro cellular crescents.

**Table 4:** Time-series change in pseudo-R^2^ values of the prognostic efficacy for renal outcomes: Max GD and individual Oxford MEST-C components.

Years	Max GD	Oxford M	Oxford E	Oxford S	Oxford T	Oxford C	Oxford MEST-C	Oxford MEST-C with Max GD
**2Y**	0.2966	0.1380	0.1079	0.0260	0.0392	0.0212	0.2778	0.5017
**3Y**	0.2966	0.1380	0.1079	0.0260	0.0392	0.0212	0.2778	0.5017
**4Y**	0.3438	0.1598	0.1249	0.0301	0.0307	0.0246	0.3384	0.6011
**5Y**	0.3438	0.1598	0.1249	0.0301	0.0307	0.0246	0.3384	0.6011
**6Y**	0.2758	0.0819	0.0021	0.0405	0.1001	0.0000	0.2299	0.5242
**7Y**	0.1432	0.0520	0.0230	0.0448	0.0962	0.0011	0.2279	0.3655
**8Y**	0.1432	0.0520	0.0230	0.0448	0.0962	0.0011	0.2279	0.3655
**9Y**	0.1432	0.0520	0.0230	0.0448	0.0962	0.0011	0.2279	0.3655
**10Y**	0.0828	0.0259	0.0094	0.0469	0.0727	0.0001	0.1592	0.2509
**End**	0.0994	0.0201	0.0086	0.0510	0.0772	0.0011	0.1593	0.2523

Abbreviations: Max GD, maximal glomerular diameter; M, mesangial hypercellularity; E, endocapillary hypercellularity; S, segmental glomerulosclerosis; T, tubular atrophy/interstitial fibrosis; C, cellular/fibro cellular crescents; Y, year: End, end of the study.

## Supplementary Materials

The following are available online at https://www.mdpi.com/2077-0383/8/12/2105/s1, Figure S1: Patient selection flowchart, Table S1: Patient clinical and laboratory characteristics, according to baseline Max GD levels (propensity score-matched cohort), Table S2: Patient histological characteristics, according to baseline Max GD levels (propensity score-matched cohort), Material S1: Supplementary Methods.
