# Supplementary material for: Maximum Glomerular Diameter and Oxford MEST-C Score in IgA Nephropathy: The Significance of Time-Series Changes in Pseudo-R2 Values in Relation to Renal Outcomes
Source: J Clin Med. 2019 Dec 2;8(12):2105. doi: 10.3390/jcm8122105 (PMC6947203; doi:10.3390/jcm8122105)
Supplement: Supplementary file 1 [file jcm-08-02105-s001.pdf]

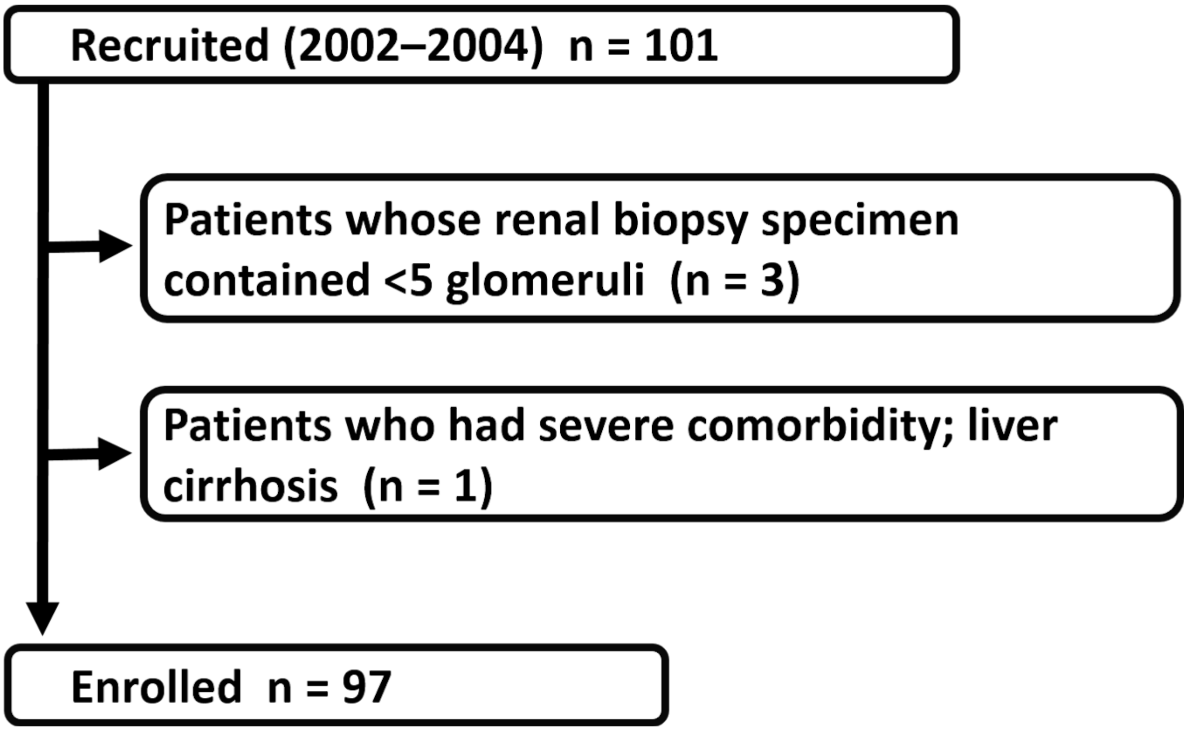

Figure S1. Patient selection flowchart. From 101 initially screened patients, three whose renal biopsy specimens contained <5 glomeruli, and one who had liver cirrhosis were excluded; the remaining 97 patients were enrolled in this study.

Supplementary Methods

Measurements of the Covariates

The baseline parameters assessed in this study were age, sex, the systolic blood pressure (SBP), diastolic blood pressure (DBP), mean blood pressure (MBP), pulse pressure (PP), body mass index (BMI), the estimated glomerular filtration rate (eGFR), the hemoglobin, serum albumin, creatinine, blood urea nitrogen, uric acid, total cholesterol, triglyceride (TG), immunoglobulin G (IgG), immunoglobulin A (IgA), immunoglobulin M (IgM), 50% hemolytic complement activity (CH50), complement component 3 (C3), complement component 4 (C4), hemoglobin A1c levels, urinary N-acetyl-beta-D-glucosaminidase (U-NAG), urinary β2-microglobulin (U-β2MG), urinary protein excretion (U-Prot), and urinary red blood cells (U-RBC). All of the biochemical analyses were performed on samples obtained after overnight fasts. The serum creatinine levels were measured enzymatically. The eGFR for Japanese patients was calculated using a previously described formula (1).

Definition of Comorbidities

The impacts of concomitant agent use and comorbidities at baseline were also assessed (2). The comorbidities were recorded as positive according to the criteria described next. Hypertension was defined as BP ≥140/90 mmHg and/or taking an antihypertensive agent. Hypercholesterolemia was defined as a serum total cholesterol level ≥220 mg/dL and/or taking an antidyslipidemic agent. Hypertriglyceridemia was defined as serum TG level ≥150 mg/dL and/or taking an antidyslipidemic agent. Hyperuricemia was defined as serum uric acid level ≥6 mg/dL and/or taking an antihyperuricemic agent. We also assessed initial treatment regimens, defined as a treatment performed during the first year after renal biopsy.

The Histological Assessment of the Kidney Biopsies

The renal biopsies from all patients were firstly assessed by an experienced nephropathologist (K.H.) before enrollment into the present study. Available renal biopsies were retrieved and analyzed for scoring the current Oxford classification of IgAN and for measuring the Max GD. The T-score was assessed by visual estimation of the percentage of the cortical area involved. The scoring of Oxford scores was performed by one examiner (T.M.) under the guidance of a nephropathologist (K.H.). The Max GD was measured by H.K. Both H.K. and T.M. were blinded to clinical data and previous nephropathologists' reports.

Supplementary References

1. Matsuo S, Imai E, Horio M, Yasuda Y, Tomita K, Nitta K, Yamagata K, Tomino Y, Yokoyama H, Hishida A, Collaborators Developing J: Revised Equations for Estimated GFR From Serum Creatinine in Japan. *American Journal of Kidney Diseases,* 53**:** 982-992, 2009
2. Ording AG, Sorensen HT: Concepts of comorbidities, multiple morbidities, complications, and their clinical epidemiologic analogs. *Clinical epidemiology,* 5**:** 199-203, 2013

Supplementary Table S1. Patient characteristics, according to baseline Max GD levels. Clinical and laboratory findings; Propensity score-matched cohort, n = 30.

Variables	Overall	Max GD ≥245.9 μm	Max GD <245.9 μm	*p*-value	Standardized Differences
	n = 30	n = 15	n = 15		
Clinical Findings					
Age (years)	40.2 ± 12.1	40.5 ± 13.9	39.9 ± 10.6	0.8835	0.049
Sex (Men; n (%))	15 (50)	9 (60)	6 (40)	0.4661	0.408
BMI (kg/m^2)	22.1 ± 3.2	21.7 ± 2.7	22.5 ± 3.6	0.5404	0.251
SBP (mmHg)	126.2 ± 16.9	126.5 ± 18.7	125.8 ± 15.5	0.9161	0.041
DBP (mmHg)	79.8 ± 13.1	78.9 ± 14.7	80.7 ± 11.8	0.7240	0.135
MBP (mmHg)	95.3 ± 13.7	94.8 ± 15.5	95.7 ± 12.2	0.8559	0.065
PP (mmHg)	46.4 ± 9.8	47.6 ± 9.4	45.2 ± 10.5	0.5137	0.241
Laboratory Findings					
Total protein (g/dL)	6.73 ± 0.68	6.79 ± 0.83	6.68 ± 0.52	0.6768	0.159
Serum albumin (g/dL)	3.85 ± 0.43	3.88 ± 0.49	3.83 ± 0.38	0.7394	0.114
Blood urea nitrogen (mg/dL)	16.9 ± 5.7	16.2 ± 4.3	17.7 ± 6.9	0.4786	0.261
Serum creatinine (mg/dL)	1.04 ± 0.31	1.03 ± 0.30	1.05 ± 0.33	0.8907	0.063
eGFR (mL/min/1.73m^2)	60.7 ± 18.7	60.5 ± 18.8	60.8 ± 19.3	0.9712	0.016
Uric acid (mg/dL)	5.99 ± 2.02	6.18 ± 1.79	5.80 ± 2.27	0.6144	0.186
Total cholesterol (mg/dL)	198 ± 35.3	197.7 ± 31.3	198.3 ± 40.1	0.9639	0.017
Triglyceride (mg/dL)	141.4 ± 81.4	155.9 ± 102.6	126.9 ± 52.6	0.3392	0.356
Hemoglobin A1c (NGSP) (%)	5.48 ± 0.31	5.50 ± 0.31	5.45 ± 0.33	0.6984	0.156
IgG (mg/dL)	1200.5 ± 252.8	1195.2 ± 268.9	1205.8 ± 244.9	0.9109	0.041
IgA (mg/dL)	341.3 ± 115.5	370.7 ± 111.8	311.9 ± 115.2	0.1667	0.518
IgM (mg/dL)	122.9 ± 58.2	111.3 ± 49.8	134.5 ± 65.1	0.2837	0.400
CH50 (mg/dL)	41 ± 8.3	40.7 ± 8.2	41.3 ± 8.7	0.8493	0.071
C3 (mg/dL)	103 ± 19.7	104 ± 23.8	101.9 ± 15.3	0.7700	0.105
C4 (mg/dL)	25.2 ± 5.8	26.4 ± 6.4	24.1 ± 5	0.2663	0.400
IgA/C3 ratio	3.33 ± 1	3.62 ± 1.08	3.03 ± 0.84	0.1018	0.610
U-Prot (g/day)	0.94 (0.50–1.57)	0.90 (0.45–1.67)	1.02 (0.55–1.34)	0.7243	0.201
U-RBC (counts/HPF)	30 (1–50)	10 (1–50)	30 (5–100)	0.2467	0.457
U-NAG (U/g・Cre)	9.59 ± 6.09	9.97 ± 6.60	9.09 ± 5.68	0.7532	0.143
U-β2MG (μg/g・Cre)	496.3 ± 832.5	704 ± 1105.9	288.7 ± 379.9	0.2762	0.502
Treatments					
Corticosteroids (n (%))	17 (56.7)	8 (53.3)	9 (60)	1	0.136
Tonsillectomy (n (%))	7 (23.3)	3 (20)	4 (26.7)	1	0.159
Corticosteroids combined with tonsillectomy (n (%))	5 (16.7)	2 (13.3)	3 (20)	1	0.181
Immunosuppressants (n (%))	2 (6.7)	2 (13.3)	0 (0)	0.4828	0.554
Antihypertensive agents (n (%))	24 (80)	12 (80)	12 (80)	1	0
ARB and or ACEI (n (%))	24 (80)	12 (80)	12 (80)	1	0
Ca blocade (n (%))	8 (26.7)	4 (26.7)	4 (26.7)	1	0
Anti-platelet agents	21 (70)	10 (66.7)	11 (73.3)	1	0.144
Anti-coagulation	2 (6.7)	1 (6.7)	1 (6.7)	1	0
EPA (n (%))	12 (40)	7 (46.7)	5 (33.3)	0.7104	0.276
No therapy (n (%))	0 (0)	0 (0)	0 (0)	-	-
Comorbidities					
Hypertension (n (%))	24 (80)	12 (80)	12 (80)	1	0
Hyperuricemia (n (%))	13 (43.3)	8 (53.3)	5 (33.3)	0.4621	0.412
Hypertriglyceridemia (n (%))	16 (53.3)	7 (46.7)	9 (60)	0.7152	0.269
Hypercholesterolemia (n (%))	14 (46.7)	6 (40)	8 (53.3)	0.7152	0.269

Continuous variables were expressed as means ± standard deviation or median (interquartile range). Count data were expressed as n (%). Abbreviations: n, number; %, percentages; BMI, body mass index; SBP, systolic blood pressure; DBP, diastolic blood pressure; MBP, mean blood pressure; PP, pulse pressure; eGFR, estimated glomerular filtration rate; IgG, immunoglobulin G; IgA, immunoglobulin A; IgM, immunoglobulin M; CH50, 50% hemolytic complement activity; C3, complement component 3; C4, complement component 4; U-Prot, Urinary protein excretion; U-RBC, urinary red blood cells; HPF, high-power field; U-NAG, urinary N-acetyl-beta-D-glucosaminidase; U-β2MG, urinary β_2-microglobulin; ARB, angiotensin receptor blocker; ACEI, angiotensin-converting enzyme inhibitor; Ca, Calcium; EPA, eicosapentaenoic acid; Max GD, maximal glomerular diameter; NA, not applicable.

Supplementary Table S2. Histological findings; Propensity score-matched cohort, n = 30.

Variables	Overall	Max GD ≥245.9 μm	Max GD <245.9 μm	*p*-value	Standardized Differences
	n = 30	n = 15	n = 15		
Number of glomeruli	18.2 ± 11.2	20.7 ± 12.6	15.6 ± 9.3	0.2137	0.461
Global sclerosis (%)	17.4 ± 15.1	18.1 ± 15.6	16.6 ± 15.1	0.7875	0.098
Segmental sclerosis or adhesion (%)	17.8 ± 14.5	17.8 ± 10.8	17.8 ± 17.8	0.9958	0
Segmental sclerosis (%)	5.62 ± 10.2	4.93 ± 8.83	6.30 ± 11.7	0.7193	0.132
Adhesion (%)	14.8 ± 12.7	12.9 ± 10.2	16.7 ± 14.9	0.4173	0.298
Crescent (%)	10.7 ± 12.1	9.9 ± 10.2	11.5 ± 14.1	0.7141	0.130
Cellular or Fibro-cellular (%)	9.06 ± 12	7.69 ± 9.73	10.4 ± 14.2	0.5409	0.223
Fibrous (%)	1.63 ± 2.99	2.18 ± 3.74	1.09 ± 1.96	0.3271	0.365
Mesangial cell proliferation (0–3)	1.50 ± 0.68	1.47 ± 0.64	1.53 ± 0.74	0.7943	0.087
Mesangial matrix expansion (0–3)	1.53 ± 0.68	1.47 ± 0.64	1.60 ± 0.74	0.6009	0.188
Interstitial fibrosis (%)	16.8 ± 9.9	19 ± 9.3	14.7 ± 10.2	0.2355	0.441
Interstitial fibrosis (0–3)	1.22 ± 0.61	1.40 ± 0.51	1.03 ± 0.67	0.1013	0.621
Interstitial inflammation (0–3)	0.93 ± 0.64	0.87 ± 0.64	1 ± 0.65	0.5772	0.202
Arteriosclerosis (0–3)	0.94 ± 0.87	0.96 ± 0.88	0.92 ± 0.90	0.9007	0.045
Arteriolar hyalinosis (0–3)	0.91 ± 0.84	0.83 ± 0.79	1 ± 0.91	0.6097	0.200
Max GD (μm)	231.7 ± 29.4	257.3 ± 7.8	206.1 ± 18	<0.0001	3.691
Oxford Classification (n = 24)					
M1	14 (58.3)	7 (58.9)	7 (63.6)	0.6968	0.097
E1	13 (54.2)	8 (61.5)	5 (45.5)	0.6824	0.325
S1	23 (95.8)	12 (92.3)	11 (100)	1	0.408
T1	5 (20.8)	3 (23.1)	2 (18.2)	1	0.121
T2	0 (0)	0 (0)	0 (0)	NA	NA
C1	12 (50)	7 (53.9)	5 (45.5)	1	0.169
C2	3 (12.5)	1 (7.7)	2 (18.2)	0.5761	0.317

Continuous variables were expressed as means ± standard deviation or median (interquartile range). Count data were expressed as n (%). Abbreviations: M, mesangial hypercellularity; E, endocapillary hypercellularity; S, segmental glomerulosclerosis; T, tubular atrophy/interstitial fibrosis; C, cellular/fibro cellular crescents.
